# SARS-CoV-2 Subgenomic RNAs: Characterization, Utility, and Perspectives

**DOI:** 10.3390/v13101923

**Published:** 2021-09-24

**Authors:** Samuel Long

**Affiliations:** Independent Researcher, Clarksburg, MD 20871, USA; nbstwne@gmail.com

**Keywords:** SARS-CoV-2, COVID-19, subgenomic RNA, sgRNA

## Abstract

SARS-CoV-2, the etiologic agent at the root of the ongoing COVID-19 pandemic, harbors a large RNA genome from which a tiered ensemble of subgenomic RNAs (sgRNAs) is generated. Comprehensive definition and investigation of these RNA products are important for understanding SARS-CoV-2 pathogenesis. This review summarizes the recent progress on SARS-CoV-2 sgRNA identification, characterization, and application as a viral replication marker. The significance of these findings and potential future research areas of interest are discussed.

## 1. Introduction

**Figure 1:**
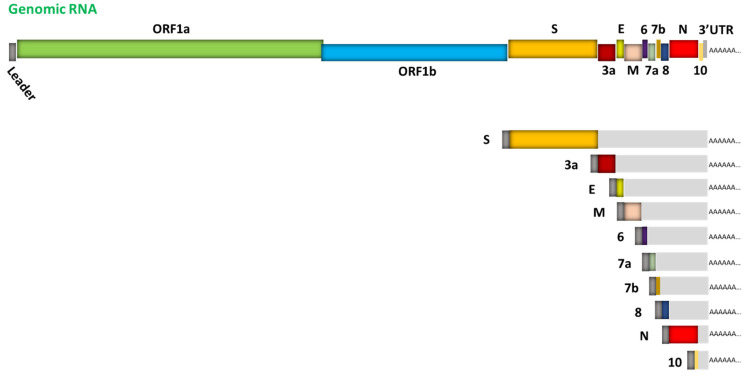
Schematic representation of SARS-CoV-2 genome organization and the canonical subgenomic mRNAs. The genome features two large genes, ORF1a and ORF1b, which encode a total of 16 non-structural proteins (nsp1-nsp16) (primary translation); structural genes encoding structural proteins include spike (S), envelope (E), membrane (M), and nucleocapsid (N), respectively; and genes encoding several small accessory proteins (3a, 6, 7a, 7b, 8 and 10). Depicted in the lower right are 10 canonical subgenomic mRNAs. Figure is adapted from [12].

**Figure 2:**
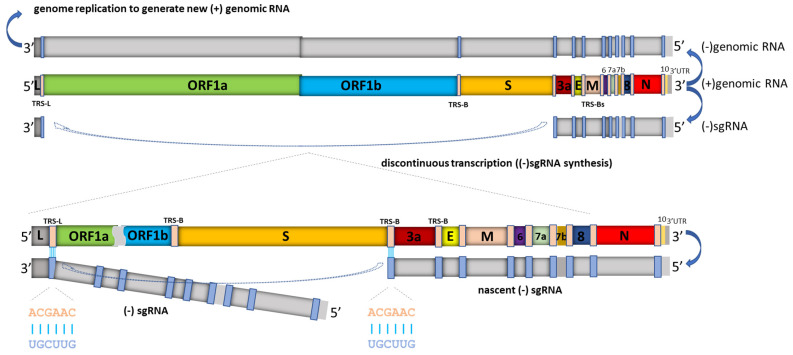
Schematic depiction of SARS-CoV-2 replication and discontinuous transcription. In addition to serving as a template for producing (−) genomic RNA (which enables genome replication), the full length (+) genomic RNA also serves as a template to produce (−) subgenomic RNAs (sgRNAs), which are subsequently used to synthesize (+) subgenomic mRNAs encoding structural and accessory proteins. (−) sgRNA synthesis involves a template switch from a body transcription regulatory sequence (TRS-B) (located upstream of most open reading frames in the 3′ one-third of the viral genome) to the leader TRS (TRS-L, located at about 70 nucleotides from the 5′ end of the genome). This discontinuous transcription process, leading to leader-body fusion, can occur at any TRS-B, and eventually results in the synthesis of a characteristic nested set of (+) subgenomic mRNAs. SARS-CoV-2 subgenomic mRNAs are structurally polycistronic, but are assumed to be functionally monocistronic [3,13], in that only the first open reading frame in each sgRNA, which is absent in the next smaller sgRNA, is translated. Depicted in the lower left and lower middle is the conserved TRS motif (ACGAAC) in the leader and body sequences. In addition, in SARS-CoV-2, extensive base pairing with 7–12 consecutive base pairs beyond the conserved motif between TRS-L and anti-TRS-B has been observed [6]. Figure is adapted from [14].

SARS-CoV-2, the etiologic agent underlying COVID-19, is a novel enveloped virus with a positive-sense, single-stranded RNA genome of about ~30k nucleotides, in the Coronaviridae family of the Nidovirales order [1,2]. Viruses in this order replicate through the transcription of negative-sense RNA intermediates that serve as templates for positive-sense genomic RNA (gRNA), and an array of subgenomic RNAs (sgRNAs), which are generated from discontinuous transcription during the synthesis of negative-strand RNA. Template switching at transcription-regulating sequences (TRS) located at the end of the “leader” sequence in the 5′ untranslated region and “body” TRS sequences located upstream of various genes in the distal third of the genome [3,4,5,6] results in sgRNAs containing a 5′ UTR “leader” sequence “fused” to the “body” sequence derived from one of the 3′ genes (Figure 1 and Figure 2).

As SARS-CoV-2 translation for most open reading frames (ORFs) (i.e., the structural/accessory ORFs) occurs via sgRNAs as the intermediates [7,8], comprehensively defining these sgRNAs is a prerequisite for the functional investigation of viral proteins, replication mechanism, and host–viral interactions involved in pathogenicity. (Since two thirds of the genome and proteins are translated from ORF1a/b, technically sgRNAs account for a minority of the viral proteins.) sgRNAs have been shown to modulate host cell translational processes [9], and it was proposed that subgenomic transcription may allow for variation in expression of the viral structural proteins and proteins involved in pathogenesis [8]. sgRNAs may also play a role in viral evolution, as template switching can cause a high rate of recombination, as observed in coronaviruses [10,11]. Several excellent reviews (e.g., [3,8,11]) provide additional information regarding sgRNA functions.

## 2. Identification of SARS-CoV-2 sgRNAs

**Figure 3:**
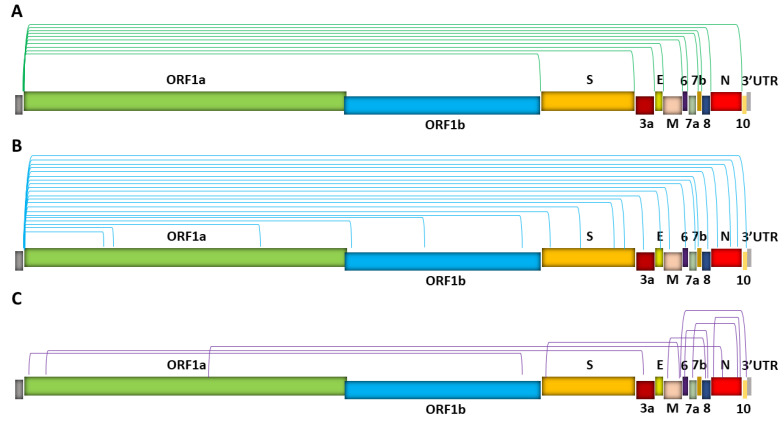
SARS-CoV-2 sgRNA recombination sites. Depicted are three types of fusion/junction sites. (Green, turquoise, or purple bracket lines represent the 5′ and 3′ locations of junctions.) (**A**) TRS-L- and TRS-B-dependent discontinuous transcription, which gives rise to canonical sgRNAs. Note that each canonical junction represents a group of subgenomes that have similar, yet distinct fusion junction sites upstream of a common first annotated gene downstream of the junction [6]. (**B**) TRS-L-dependent noncanonical fusions between TRS-L and unanticipated 3′ sites in the middle of ORFs or UTR (i.e., noncanonical 3′ sites) in the body. (**C**) TRS-L-independent fusion between sequences that share no similarity to the leader, resulting in long-distance fusions and smaller deletions mainly in the structural and accessory genes when the fusion occurs between proximal sites. Hundreds of noncanonical sgRNAs have been identified [5,6,12,20], and in (**B**,**C**), only several representative fusion patterns are illustrated. In addition, both in-frame and out-of-frame fusion products can be generated in (**B**,**C**), with out-of-frame noncanonical sgRNAs significantly outnumbering in-frame noncanonical sgRNAs (by ~60%) [12].

Using complementary DNA nanoball sequencing (DNB-seq) and nanopore direct RNA sequencing (DRS) techniques, Kim et al. [12] identified several canonical sgRNAs in SARS-CoV-2-infected Vero cells (in agreement with genomic sequence annotation [15]) that encode the conserved structural proteins S (spike protein), E (envelope protein), M (membrane protein), and N (nucleocapsid protein) and accessory proteins 3a, 6, 7a, 7b, 8, and 10, as part of a high-resolution map of the SARS-CoV-2 transcriptome and epitranscriptome. Each canonical junction represents a group of subgenomes that have similar, yet distinct fusion junctions upstream of a common first annotated gene downstream of the junction [6]. SARS-CoV-2 canonical sgRNAs were also described in several other studies [5,16,17,18]. Numerous noncanonical sgRNAs were also identified, which were a result of truncated fusions, frameshifted ORFs, and body-to-body junctions, creating a diffuse pattern of junctions across the genome [5,6,12,16,19,20] and indicating complex, discontinuous transcription events that can alter the landscape of viral open reading frames (Figure 3). The noncanonical junctions are not associated with a TRS-like homology [6,12,20].

## 3. Synthesis and Subcellular Localization

The fraction of viral to total cellular protein translation in host cells surges by as much as 20,000 fold within hours after infection by beta-coronaviruses, while over the same time period, the amount of virus positive-sense RNA increases up to 200-fold, much of which is sgRNA [21,22]. Significant depletion in intracellular glucose and folate in SARS-CoV-2-infected cells [22] suggests the possibility that host glucose and folate metabolism are hijacked to respond to the demand of viral sgRNA replication. This is accompanied by a significant decrease in host mRNA abundance, likely due to the virus’ ability to shut off host transcription to channel host nucleotide supply to viral biosynthesis [22,23,24]. A model is emerging that indicates that SARS-CoV-2 induces post-transcriptional glycolysis and one-carbon metabolism in newly infected cells; serine metabolism, particularly by serine hydroxyltransferase 1, which is implicated in the cytosolic branch of the host one-carbon metabolism, produces carbon units for de novo purine synthesis, enabling massive sgRNA and non-structural protein generation, and viral replication [22].

Using metabolic labeling of newly synthesized viral RNA followed by quantitative electron microscopy autoradiography, Snijder et al. [25] established the double membrane vesicles (DMVs) as the site of coronavirus RNA synthesis. This was supported by the presence of double-stranded RNA, the de novo synthesized RNA, and putative viral replication intermediate inside the DMV, in a cryo-transmission electron microscopy study [26]. DMVs provide a favorable environment for viral RNA replication by creating an appropriate replicase topology and a physical barrier between the viral replication compartments and the innate immune sensors and RNA degradation machinery in the cytosol [27]. Once synthesized, sgRNAs can potentially be transported into the cytosol through membrane pores, the opening action of which has been captured in a whole-cell and subcellular compartment 3D reconstruction study [28]. This same light and electron microscopy-based study also detected ribosomes on the cytosolic side of DMVs, consistent with newly synthesized viral RNAs being used for protein synthesis directly.

RNA-FISH labeling of SARS-CoV-2-infected cell cultures [29], mice [30], and patient autopsy samples [29] suggests that viral RNA is predominantly found in the cytoplasm. Computational work by Wu et al. [31], however, suggested strong preferential residency of SARS-CoV-2 sgRNAs in the mitochondria and nucleolus [31], although it is yet to be shown in a virological system that viral RNA shuttles to the mitochondria. Cortese et al. [28] observed strong perturbation of mitochondrial morphology (e.g., display swollen cristae and matrix condensation) and function in infected cells, including a drastic decrease in the mitochondrial ATP synthase subunit 5B. This, however, most likely reflects SARS-CoV-2-induced attenuation of cellular energy metabolism, stress, apoptosis, or innate immunity, and does not directly support sgRNA being localized in the mitochondria or driving dysfunction of this organelle in SARS-CoV-2-infected cells. The biology surrounding SARS-CoV-2′s impact on mitochondria-based immunity in patients with age-related conditions, such as diabetes, obesity, and dementia [32,33,34], is still very much evolving and presents ample research opportunities.

## 4. Expression and Detection

sgRNAs are detected during early symptomatic infection and in some cases after symptoms have subsided [35,36]. The detection window/duration varies widely, between 2 and 162 days after symptom onset [35,36,37,38,39,40,41,42,43,44,45], depending on factors such as the assay(s) employed, sample and tissue source [42], severity of symptoms at time of sampling [38], patient immunosuppressive status, age [43], underlying condition(s) [43], and therapy [35].

Several studies used methods that specifically detect expressed RNA to quantify the abundance of individual canonical sgRNAs in cell lines infected with SARS-CoV-2 [12,16,19,46]. Kim et al. [12] quantitatively compared the junction-spanning reads to demonstrate that subgenomic N RNA is the most abundantly expressed canonical sgRNA species, followed by S, 7a, 3a, 8, M, E, 6, and 7b. Davidson et al. [16], using an ORF-centric pipeline assessment (with different sequence inclusion criteria from [12], and possible contribution from dataset-specific factors), found sgRNAs from the M ORF are the second most abundant groups after N, and their results broadly agree with [19], and with previously published reports of the protein levels in SARS-CoV-2, with M and N showing the highest expression levels [46,47]. In all studies, ORF10 expression is consistently the lowest or absent [5,12,16,20,48]. Recently, a panel of seven sensitive RT-ddPCR-based assays was used to measure the expression of canonical sgRNAs in the pharynx of an acutely infected individual [49]. In this study, the N RNA showed the highest expression level, followed by M and 3a, while E was the lowest (6, 7b, and 10 were not studied in this report). In general, the published relative sgRNA abundance likely results from polarity in the sgRNA synthesis process, e.g., the N sgRNA is most abundant because its TRS-B is infrequently bypassed during minus RNA strand elongation.

Nomberg et al. [20] performed a junction abundance-based analysis of several independent SARS-CoV-2 transcriptomes generated using three sequencing strategies (DRS, Illumina polyA, and total RNA sequencing). Results from five host systems (Vero cells, A549 cells, Calu3 cells, bronchial organoids, and ferret nasal washings) and seven viral isolates showed that noncanonical sgRNAs constitute up to 1/3 of the total sgRNAs in cell culture infection models (and up to 1/2 in a ferret in vivo model), are generally consistent in abundance across the transcriptomes analyzed, and rise in level over time during infection. These results are consistent with the finding of [12] that the combined noncanonical sgRNA read numbers are often comparable to the levels of canonical sgRNA transcripts. Although it is well known that canonical sgRNA transcription is essential for replication, the importance of non-canonical sgRNA transcription remains to be determined. It will be important to definitively determine if noncanonical sgRNAs are actually translated and yield functional products, and to study their potential role in the viral life cycle and host immune responses, considering that defective genomes in negative-sense RNA viruses have been associated with antiviral immunity, dendritic cell maturation, and interferon production [50,51,52,53]. In experimental systems, such as in [20], where a great abundance of non-canonical RNAs was found, it seems plausible that a similar abundance of canonical and non-canonical sgRNA may indicate a similar level of importance for replication/survival or pathogenesis. Individual noncanonical sgRNA species are expressed at low levels but the number of these species is large [5]. Noncanonical sgRNAs can span a wide spectrum in length, which, in combination with individual molecules’ low abundance, can potentially explain why, in earlier literature, these molecules were not readily detectable (such as in Northern blots), as non-canonical sgRNAs were likely mistaken as background signals in such analyses.

Significant discrepancies exist regarding the estimates of sgRNA abundance relative to gRNA, depending on the analytical method. In addition, experimental systems (including sample types, e.g., infected cells vs. patient samples), how samples are collected and processed upstream of even the same quantitation method (e.g., RT-qPCR or sequencing), and the virus under study, can all potentially have a profound effect on the prevalence and/or detection of canonical and non-canonical sgRNAs. For example, earlier Northern blotting and reverse transcription PCR (RT-PCR)-based data of the transmissible gastroenteritis virus (TGEV) (another member of the Coronaviridae family) in an infected cell line (e.g., [4,54]) showed that the combined quantity of sgRNAs significantly exceeds that of gRNA, and the individual canonical sgRNA amount can approach (and in some instances be higher than) the level of gRNA. In contrast, an RT-ddPCR sgRNA assay panel analysis [49] (see above) estimated that the total canonical sgRNA species represented ~55% of the gRNA copies or ~36% of total viral RNA in an acutely infected SARS-CoV-2 patient. Worfel et al. [37], using a real-time PCR-based assay for the relatively non-abundant sgRNA, E RNA, estimates the sgRNA abundance to be only 0.4% of SARS-CoV-2 gRNA in hospitalized patient samples. SARS-CoV-2-infected cells contain positive- and negative-sense genomic and subgenomic RNA, but a cell-free culture supernatant or a clarified clinical sample likely is enriched for genomic RNA. For example, the sputum samples from the Wölfel study [37] have been clarified by centrifugation (i.e., selecting for free virus or RNA and down-selecting for infected cells or cellular debris that might contain sgRNA) prior to RNA extraction, while the Telwatte et al. ddPCR study [49] specifically and intentionally pelleted the cells in the clinical samples (nasopharyngeal swabs) to selectively isolate cell-associated RNA. The differences in processing of these clinical samples can therefore largely explain the differential results (0.4% vs. 55%) in these latter two studies. In addition, there are obvious caveats associated with the different methods to identify sgRNA, such as the target regions with differing abundance as sampled by various PCR-based assays. This will be further discussed in the “Analysis approaches” section.

As most published sgRNA abundance data are derived from cell lines, further studies with larger numbers of clinical samples are required to confirm the above findings. In addition, it will be of interest to monitor the kinetics of individual sgRNAs during disease progression in patient samples. (Clinical samples can present challenges especially for amplicon-based sequencing approaches due to sample quality limitations; however, see the “Analysis approaches” section below.)

## 5. Mutations

In most cases the SARS-CoV-2 variants in gRNA are transmitted to sgRNAs with high fidelity [5]. A variant in the spike protein, D614G (B.1 lineage), emerged early in the pandemic [43,55,56,57,58]. Various lineages from this genetic background harboring additional mutation(s) (such as a major adaptive mutation N501Y) rapidly became dominant in geographical locations where they have circulated, including UK, South Africa, Brazil, California, and India, among others (reviewed in [59]; also see below). A recent report [60] identified a novel variant within the subset of sequences harboring the D614G mutation and contains adjacent nucleotide changes affecting two residues of the nucleocapsid protein (R203K/G204R; B1.1 lineage), which have emerged by homologous recombination from the core sequence of the TRS and resulted in the generation of a novel sgRNA transcript for the C-terminal dimerization domain. This has been confirmed by deep sequencing of ~1000 clinical samples. Increased expression of other sgRNA species was detected in this new variant, in addition to a higher level of nucleocapsid proteins. The ability of SARS-CoV-2 to introduce new TRS motifs in its genome, with the potential for novel sgRNA transcripts and coding changes, suggests this as a means for diversification and adaptation in the host. This highlights the importance of continued surveillance of viral evolution and elucidation of potential functional consequences (e.g., on pathogenicity and/or transmission) of newly emerged genetic changes in guiding the development of diagnostics, antivirals, and universal vaccines.

## 6. RNA–RNA Interactions

Host–virus RNA–RNA interactions have been reported to regulate the replication of some RNA viruses [61,62,63]. Utilizing a method crosslinking matched RNAs and deep sequencing for in-depth RNA conformation capture (COMRADES) in SARS-CoV-2-infected living cells, Ziv et al. [64] identified site-specific interactions between viral sgRNAs and a variety of cellular RNA, including small nuclear RNAs (snRNAs) and long cellular RNAs. Interestingly, one of the long cellular RNAs, the host ribonuclease MRP RNA, which base pairs extensively with SARS-CoV-2 sgRNAs, has been implicated in viral RNA degradation [65] as well as human pre-ribosomal RNA processing [66], consistent with sgRNA also potentially regulating host cell translational process (i.e., bidirectional modulation between host and virus). In addition to host–virus RNA–RNA interactions, this study also revealed networks of RNA–RNA interactions (i.e., both short- and long-range) that span the entirety of the viral gRNA and sgRNAs. Some of the long-range interactions are potentially involved in regulation of discontinuous transcription, as they locate cis-elements that can interact to generate genome topologies conducive to the synthesis of the sgRNA series.

## 7. Utility in Clinical and Research Settings

sgRNA-specific qPCR assays (across leader-body junctions) have been widely used to measure replicating SARS-CoV-2 in both human patients and animal models [37,38,67,68]. Wolfel et al. [37] was among the first reports that detected active virus replication in the throat of hospitalized patients by virtue of the presence of viral replicative RNA intermediates (based on the subgenomic E RNA assay). This finding had important implications for COVID-19 containment. Another frequently used assay is based on subgenomic N RNA, which is transcribed at a significantly higher level than subgenomic E RNA. Primers designed in the nucleocapsid are used in most clinical qRT-PCR assays; consequently, detection of nucleocapsid sgRNA has been a major facet of SARS-CoV-2 clinical testing and public health efforts. Collectively, these two assays have been used to pinpoint the cellular targets of viral tropism and replication in patient lungs and airways, and show direct viral infection in vascular endothelial cells [43]. The assays have also allowed improving the diagnosis of hospitalized patients through testing stool samples (especially in patients suspected of being infected, but with negative upper respiratory tract viral RNA results) [69], and inferring active viral replication in cases with prolonged persistent SARS-CoV-2 RT-PCR signals [45].

Persistent infection of SARS-CoV-2 in immunocompromised individuals has also been of significant concern [40,41,42,70,71], as such hosts could serve as reservoirs for mutation accumulation and new viral strains capable of evading immune responses elicited during the course of infection or induced by vaccine. At least one SARS-CoV-2 variant/lineage may have resulted from long-term replication in an immunocompromised host, especially with the lack of closely related viral isolates [72]. In three pediatric and young adult patients, there was convincing evidence (based on a combination of sgRNA and viral cultural analysis) of ongoing replication and viral infectivity for up to 162 days since the initial detection of an infection [42]. Interestingly, complementary sequencing analysis revealed mutations in several regions within the spike gene, including in residues and regions whose mutations have been implicated in enhanced infectivity [73], abolishment of the binding of the anti-spike protein 4a8 blocking/neutralizing monoclonal antibody [70,74], conferring antibody escape [75], enhancing affinity of the binding of the spike protein to the ACE2 receptor [76], and associated with the South Africa S.501Y.V2 lineage [75,77,78,79]. It is noteworthy that similar mutations (e.g., N440D, E484A, and E484K) have independently emerged in other immunocompromised patients who were persistently infected [41,70]. These findings highlight the necessity of genomic surveillance [72,80] and implementing infection control precautions in the management and care of immunocompromised pediatric and young adult population and immunocompromised patients in general.

Several important studies for understanding SARS-CoV-2 pathogenesis and transmission dynamics and assessing the efficacy of vaccines and therapeutics have been conducted in clinically relevant non-human primate (NHP) models, such as rhesus macaques, cynomolgus monkeys, and African green monkeys [81,82,83,84,85,86,87,88]. These models have distinct advantages over human subjects, including ease of control over experimental variables and ability for repeated sampling, among others [89,90]. As a respiratory virus, SARS-CoV-2 presents unique challenges in these animals, as preclinical studies typically introduce viral challenges in the respiratory tracts (i.e., via the intranasal and intratracheal routes), while infection monitoring post-challenge uses samples from the same anatomical locations. Under such study scenarios, assays based on a total RNA or viral gRNA target would recognize both input challenge and newly replicating viruses, and would not permit measuring protective efficacy or drug effects, especially at early time points. An sgRNA-specific assay enabled quantifying a replicating virus in several important NHP vaccine/challenge studies ([67,68,82]; testing the efficacy of mRNA-1273, ChAdOx1 nCoV-19, and Ad26 vaccines, respectively), and evaluating the protective efficacy of natural immunity and mAbs in NHP models [82,91]. These results collectively highlight the utility of sgRNA in studies investigating the prophylactic and therapeutic efficacy of vaccines, mAbs, and antivirals in NHP models.

Truong et al. [42] recently reported overall good correlation between detection of viral intermediates and viral culture data, suggesting sgRNA may serve as a convenient molecular surrogate for infectivity as well. Speranza et al. [81] directly demonstrated that in tissues, sgRNA is a more sensitive detection method than virus isolation in tissue culture, likely due to the culturing methods’ limitation of sample quality.

## 8. Analysis Approaches

Several methods, each with unique strengths and limitations, have been used to identify sgRNA (Table 1). For example, Northern blotting can provide information about sgRNA size and sample integrity, but it is time-consuming and suffers from low sensitivity. In addition, like all hybridization-based approaches, Northern-blotting can introduce a high background resulting from cross-hybridization, which contributes to a limited dynamic range of detection (due to both background and saturation of signals). Reverse-transcriptase PCR (RT-PCR) is a semi-quantitative, faster, and more sensitive alternative to Northern blotting. Real-time PCR is widely accepted, and is least time intensive and technically demanding, with benefits such as a large dynamic range, single-copy signal detection sensitivity, no post-amplification processing, and a relatively high throughput. However, in the context of an sgRNA abundance study, quantification results of PCR products generated from primer pairs designed against different regions of the genome require careful interpretation. In addition, Northern, RT-PCR, and real-time PCR share the same major limitation in that they require previous knowledge of the RNA molecules to be analyzed, therefore limiting the potential for discovery. The next generation sequencing (NGS) method is a hypothesis-free approach that does not require known sequence information; provides the discovery power to detect novel genes and rare variants; and quantifies transcripts in a high throughput fashion. Admittedly, NGS procedures are significantly more complicated than real-time PCR, and reproducibility can present an issue due to the complexity of NGS experiments [92]. One main limitation of NGS is in the area of quantifying low copy number templates (including low-abundance sgRNA species). Due to the random sampling nature of NGS, its sensitivity is largely determined by “sequencing depth” (i.e., transcripts expressed at low levels may not reach the necessary depth to yield reads). It is well known that, for low copy number transcripts, the correlation between NGS and real-time PCR has been relatively poor [93]. Further, in NGS/RNA-seq, some regions (such as GC-rich regions) may be more difficult to process and are subsequently underrepresented. In addition, at the NGS/RNA-seq data analysis step, normalization assumptions and parameters in reads mapping algorithms (such as the mismatch allowance setting) can also significantly impact results. Due to the above considerations, a frequently used approach utilizes NGS to discover and narrow down molecules of interest, and then relies on qPCR to verify gene expression, especially when the template copy numbers are low.

Limited yields of cells or fluids from sampling procedures such as nasopharyngeal swabs, and the presence of potential inhibitors (e.g., chemical or protein contaminants) in clinical samples, require that PCR amplification and detection be highly sensitive and reliable during SARS-CoV-2 nucleic acid analysis. Digital PCR has demonstrated significant advantages in both SARS-CoV-2 gRNA [94,95,96] and sgRNA [49,97,98] studies due to its ability for absolute quantification [98,99,100,101,102,103,104,105], tolerance to inhibitors [106], increased precision at low analyte copy numbers [107,108,109], and inter-run reproducibility [110,111,112]. One additional distinct advantage of the digital PCR approach is its lower susceptibility to sequence mismatches, which is especially relevant as emerging mutations that can potentially predominate could affect the performance of real-time PCR-based assays if they occur in regions where the PCR primer and probes are located [113,114]. For example, Penarrubia et al. [115] found that up to 34.4% of SARS-CoV-2 genomes contain mutation(s) capable of affecting PCR primer annealing in published real-time PCR assays.

Various sequencing strategies (including deep sequencing and direct RNA sequencing) were attempted to comprehensively characterize the spectrum of SARS-CoV-2 sgRNAs in cell lines and patient samples [12,36,37]. Commonly used whole genome targeted sequencing methods typically employ pairs of primers to generate cDNA amplicons for downstream sequencing. This approach imposes constraints on the primer locations and amplicon numbers and cannot resolve the RNA junctions not flanked by primer pairs. A recently described Tiled-ClickSeq approach used a tiled primer-based single reverse transcription reaction to eliminate the need for paired primers, as the other end of the cDNA is generated by azido-nucleotides that terminates cDNA synthesis stochastically [116]. This approach employs hundreds of tiled primers along the virus genome to simultaneously characterize sgRNAs and other variants, and provides a robust platform that analyzes the full range of RNA species in one simple assay. In addition, Doddapaneni et al. [48] used oligonucleotide capture enrichment followed by deep short-read sequencing to achieve uniform target coverage of gRNA and sgRNAs. This latter method performs especially well with clinical samples containing degraded source material [117,118,119], compared to amplicon-based sequencing approaches.

Being a relatively fast and cheap method to simultaneously quantify multiple targets, PCR-based assay panels [102,120,121,122] provide the resolution required to decipher the apparent complex viral transcription dynamics over the course of infection. A panel of sensitive, digital PCR-based assays targeting multiple SARS-CoV-2 sgRNAs was described [49,97]. In addition, a strategy that targets multiple regions can have a higher sensitivity in sgRNA detection. Penarrubia et al. [115] used a panel of real-time PCR assays to reduce the signal detection loss associated with new genomic variants in single assay analysis. As proposed by Telwatte et al. [49,97], assay panel analysis can enable measuring and discriminating the discontinuous transcription rates at various loci and differentiate among the abundance of different sgRNAs within the same sample. Such results can be used to assess the potential correlation between the sgRNA levels and parameters such as disease severity and viral infectivity/transmission, shedding light on the SARS-CoV-2 transcription kinetics and regulatory mechanisms during the infection course.

As briefly discussed above, results in studies [12,16,19,20,21] describing the canonical and non-canonical transcripts can be significantly impacted by experimental systems and upstream sample processing (including how samples are collected and stored). For example, viral RNA species assessed in infected cell cultures such as Vero (i.e., infected cells containing positive- and negative-sense genomic and subgenomic RNA) are likely to be different than those in nasal washes (containing free infectious and non-infectious virus, sloughed infected epithelial cells with positive- and negative-sense viral RNA, and virus particles in complex with antibody/host protein). Similarly, sample type presumably will also have a major impact on preclinical studies, such as those carried out in the NHP models as described above (e.g., a nasal wash vs. lung tissue should give different results when measuring sgRNAs). Legitimate questions along this line include, for example, is the SARS-CoV-2 transcriptome or epitranscriptome different in a monkey kidney cell line (Vero) versus in epithelial cells in the lung or even human primary airway epithelial cells in culture? Is it possible that the high level of replication in Vero cells could create rare artifacts detected by sensitive and powerful sequencing techniques that are even more rare or not found in nature? Additionally, different computational methods have the potential to yield different results in RNAseq analysis using the same data set [12,16,19,20]. Due to the above considerations, it may be worth re-examining the non-canonical sgRNA concordance data among different types of samples.

## 9. Perspectives and Conclusions

Additional mechanistic studies regarding SARS-CoV-2 sgRNAs are needed. Findings regarding SARS-CoV-2 sgRNA biogenesis from cell lines need to be reevaluated in infected primary cells or patient tissue samples under physiological conditions, and results derived from bioinformatics (such as regulatory features governing template switches as predicted by computational RNA–RNA base pairing [6] and residency signals [31]) should be verified by experiments. The SARS-CoV-2 sgRNA coding regions can be examined via polysome profiling (e.g., with solid state nanopores) to assess the ribosome footprints and determine which parts of the subgenomes are translated. In addition, extensive SARS-CoV-2 sgRNA modifications have been identified based on ionic current differences between an unmodified synthetic template and viral transcripts in nanopore DRS [12], and provide an additional potential layer of SARS-CoV-2 regulation. It will be of interest to study the chemical nature, regulation, and biological functions of these modifications.

One important unresolved issue concerns the mechanism that allows for persistence of the sgRNAs [41,42]. The transcription of gRNA and sgRNA occurs at DMVs that contain cellular and viral materials in the cytoplasm of infected cells [25,26,123]. While the gRNAs are packaged into virions, it seems unlikely that the sgRNAs are [35]. Therefore, sgRNA is considered a suitable marker for active infection as it presumably degrades more rapidly than gRNA. However, there remains the possibility that at least a fraction of the sgRNAs are protected from nuclease degradation by encapsulation in DMVs and/or extracellular vesicles even after replication has ceased [36]. Delineating the contribution of “lingering” sgRNA to total sgRNA signal will bear significantly on the interpretation of the clinical significance (i.e., duration of infectivity) of several reports indicating prolonged sgRNA detection in patients [40,41,42,45] and on the efficacy results from the NHP animal vaccine/challenge studies as described above [67,68,82,91].

One research area of potential interest is the noncanonical sgRNAs [13]. These diverse fusion transcripts were found in both DRS and DNB-seq, and the expression of some of them were verified by RT-qPCR [12]. The combined noncanonical sgRNA sequence reads can be similar in number to that of accessory transcripts [12,20]. Importantly, most of these RNAs have coding potential and their products can be truncated versions of known accessory proteins or proteins distinct from known viral proteins. In fact, Davidson et al. [16] recently provided peptide mapping evidence from tandem mass spectrometry, indicating the detection of previously unknown viral proteins, a fraction of which could be of noncanonical sgRNA origin. Based on the unique recombination sequences observed in noncanonical sgRNAs, it has also been suggested that they may function as defective interfering RNAs—subviral RNAs generated by the error-prone viral replicase in the process of RNA virus multiplication (i.e., parasitic RNAs derived from parent virus and depending on viral-coded protein factors for multiplication) [6,124]. As postulated by Kim et al. [12] and Nomburg et al. [20], noncanonical sgRNAs with the 5′ end of ORF1a may modulate the relative abundance of nonstructural viral proteins. These will be important topics for future studies. In addition, it will be of interest to understand the factors and mechanism(s) that control the formation of noncanonical sgRNAs.

Important work regarding the functions of sgRNAs in other coronaviruses has been published. In addition to their critical role in generating several structural and accessory proteins encoded in the 3′ region of the viral genome [125,126,127], sgRNAs’ part in the life cycle and pathogenicity of coronaviruses has also been well-documented [128,129,130,131,132]. sgRNAs may function as important mediators of positive-strand synthesis [133]. In addition, high levels of sgRNA redundancy were detected in members of the order Nidovirales, which function to ascertain continued protein synthesis when regulatory sequences are mutated [134]. Interestingly, the AAGAA-type modification clusters identified on sgRNAs [12] may promote viral survival and facilitate immune evasion [135]. The fact that sgRNAs in other viruses play roles in viral replication and recombination warrants investigating similar roles of sgRNAs in SARS-CoV-2. Li et al. [136] recently observed the presence of a nsp15 (a nidoviral RNA uridylate-specific endoribonuclease) cleavage site in the TRS motif, pointing to a possible negative feedback mechanism of regulating SARS-CoV-2 transcription and replication via controlling the relative proportion of sgRNAs and gRNAs. Given our limited understanding concerning the clinical implications of SARS-CoV-2 sgRNAs, much remains to be studied to uncover the role they play in pathogenicity, the mechanism(s) through which they function, and their potential as therapeutic targets.

## Figures and Tables

**Table 1:** Summary of sgRNA analysis approaches. The strengths and limitations of each analysis approach are listed.

Approach	Strengths	Limitations
Northern blotting	Provides information regarding sgRNA size Provides information regarding sample integrity	Time consuming Low sensitivity Limited dynamic range of detection Limited potential for discovery
Reverse transcription PCR	Faster, more sensitive than Northern blotting	Only semi-quantitative Limited potential for discovery
Real time PCR (including panels)	Large dynamic range Single copy detection sensitivity Technically simple (no post-amplification processing) Relatively high throughput Least time consuming Assay panels can discriminate the discontinuous transcription rates at various loci	Quantification results can vary based on assay design region Limited potential for discovery
Digital PCR (including panels)	Ability for absolute quantification Tolerance to inhibitors Increased precision at low analyte copy numbers and high inter-run reproducibility Lower susceptibility to sequence mismatches Panels have increased sensitivity in sgRNA detection relative to single assays	Limited potential for discovery
Next generation sequencing (NGS)	Provides the power to discover new species High throughput quantification of transcripts	Complicated procedure/workflow and related reproducibility issue Limited ability to quantify low abundance sgRNA species Uneven representation of some sequences Results can vary depending on data analysis parameters Amplicon-based approaches cannot resolve certain RNA junctions Amplicon-based approaches have compromised performance on degraded source material

## Data Availability

All data generated or analyzed during this study are included in this published article.
