# Correction: Long, S. SARS-CoV-2 Subgenomic RNAs: Characterization, Utility, and Perspectives. *Viruses* 2020, *13*, 1923

**DOI:** 10.3390/v14071406

**Published:** 2022-06-28

**Authors:** Samuel Long

**Affiliations:** Independent Researcher, Clarksburg, MD 20871, USA; nbstwne@gmail.com

## Affiliation Information

The author is no longer affiliated with the original institution “AIDS and Cancer Virus Program, Frederick National Laboratory for Cancer Research, Frederick, MD 21702, USA”, so the author wishes to change the affiliation to “Independent Researcher, Clarksburg, MD 20871, USA”. The telephone number should be removed in regard to the change of affiliation.

## Funding Information

The author himself paid the publication charges, therefore in the funding information section, the author wishes to remove the following statement in the original article [1], “Funded with Federal funds from the National Cancer Institute, National Institutes of Health, under contract numbers HHSN261200800001E and 75N91019D00024.”, and replace it with the following statement, “This research received no external funding”.

The author apologizes for any inconvenience caused and states that the scientific conclusions are unaffected. This correction was approved by the Academic Editor. The original publication has also been updated.
